# Cadmium-109 Radioisotope Adsorption onto Polypyrrole Coated Sawdust of *Dryobalanops aromatic*: Kinetics and Adsorption Isotherms Modelling

**DOI:** 10.1371/journal.pone.0164119

**Published:** 2016-10-05

**Authors:** Michael Adekunle Olatunji, Mayeen Uddin Khandaker, Yusoff Mohd Amin, Habibun Nabi Muhammad Ekramul Mahmud

**Affiliations:** 1 Applied Radiation Laboratory, Department of Physics, University of Malaya, Kuala Lumpur, Malaysia; 2 Polymer Laboratory, Department of Chemistry, University of Malaya, Kuala Lumpur, Malaysia; Duke University Marine Laboratory, UNITED STATES

## Abstract

A radiotracer study was conducted to investigate the removal characteristics of cadmium (^109^Cd) from aqueous solution by polypyrrole/ sawdust composite. Several factors such as solution pH, sorbent dosage, initial concentration, contact time, temperature and interfering metal ions were found to have influence on the adsorption process. The kinetics of adsorption was relatively fast, reaching equilibrium within 3 hours. A lowering of the solution pH reduced the removal efficiency from 99.3 to ~ 46.7% and an ambient temperature of 25°C was found to be optimum for maximum adsorption. The presence of sodium and potassium ions inhibited ^109^Cd removal from its aqueous solution. The experimental data for ^109^Cd adsorption showed a very good agreement with the Langmuir isotherm and a pseudo-first order kinetic model. The surface condition of the adsorbent before and after cadmium loading was investigated using BET, FESEM and FTIR. Considering the low cost of the precursor’s materials and the toxicity of ^109^Cd radioactive metal, polypyrrole synthesized on the sawdust of *Dryobalanops aromatic* could be used as an efficient adsorbent for the removal of ^109^Cd radioisotope from radionuclide-containing effluents.

## Introduction

Environmental pollution as a result of the frequent discharge of industrial waste effluents into land and seawaters has become a global challenge particularly in the 21st century. This is due to the presence of various pollutants in these aqueous wastes, which could lead to harmful health effects on both human and animal lives. Due to their non-biodegradability, toxicity and hazardous nature, cadmium and its radioisotopes have been classified as dangerous to health [1,2]. Once it gets into human body via the food chain, cadmium can cause health dysfunctions such as itai-itai [3,4]. Cadmium has nine major radioactive isotopes, of which only three: cadmium-109, cadmium-113 and cadmium-113m have half-lives long enough to warrant potential concern. Among them, ^109^Cd is a short-lived radioisotope (T_1/2_ = 1.3 yrs), and once produced, it decays to the stable ^109^Ag via an EC+β^+^ process followed by the emission of energetic gamma-rays (Eγ = 88.0336 keV; Iγ = 3.70%). So, when it is dispersed into the environment from any sources, its activity will be remaining for at least 10 yrs (~ equal to 8 half-lives), and thus considered as hazardous. Moreover, the very high specific activity (2600 Ci/g) of ^109^Cd made it more concern for health when ingested. Accordingly, different regulatory frameworks are implemented to reduce consumption of cadmium from various environmental samples following their contamination levels and locations [3].

Among the various techniques of metal ions removal from aqueous solutions, adsorption is considered the most feasible and affordable [5]. This is due to the availability of low cost adsorbents and/or adsorbent precursors, simple operational requirements and performances [6,7]. In addition, adsorption shows superiority in wastewater treatment due to its insensitivity to toxic pollutants, and does not result in the formation of harmful substances [8]. On the other hand, among the two widely used conducting polymers (polypyrrole and polyaniline), polypyrrole shows some favorable features (e.g., simple doping chemistry, simple metal adsorption-desorption mechanism and relative air stability) for practical applications [9,10]. However, the preparation conditions as well as the sizes of the dopants plays a significant role in the sorption capacity of polypyrrole [11].

A review of literature showed that the polypyrrole conducting polymer has been synthesized and used for the removal of fluoride ions [12], lead ions [13] and other metal ions [14,15]. In recent time, incorporation or coating of polypyrrole on appropriate media has been discovered as a novel approach to enhance the sorption capacity of polypyrrole, and this has resulted in the higher efficiency of this polymer for adsorption of heavy metal ions. Graphene oxide, activated carbon and wood sawdust are some of the reported materials that can be used to modify polypyrrole for better sorption of heavy metal ions [9,16–18]. Considering the cost implications of most materials, sawdust appears to have an advantage over others due to its abundance, low cost, and possession of natural binding force for pollutants [16].

Recognizing such favorable features, we synthesized polypyrrole on the sawdust of *Dryobalanops aromatic* by chemical polymerization using iron (III) chloride hexahydrate (FeCl_3_.6H_2_O) as oxidant. Polypyrrole synthesized with FeCl_3_.6H_2_O possesses releasable dopants, such as Cl^-^, that could easily exchange with Cd ions in the aqueous medium. The choice of wood sawdust is due to the fact that it has some reactive functional groups which could enhance those in polymers leading to better adsorption efficiency. Specifically, efficiency of the polymer composite for Cd adsorption was investigated as a function of solution pH, temperature and concentration. Also, the kinetic and isotherm parameters using different models were determined for practical applications.

## Materials and Methods

### 2.1 Chemicals and Materials

All chemicals used were analytical reagent grade. FeCl_3_.6H_2_O, sodium nitrate (NaNO_3_) and potassium nitrate (KNO_3_) salts, NaOH and HCl were obtained from Merck AG (Germany) and pyrrole (98%) from Acros. All chemicals and materials were used as obtained from the suppliers without further purification except for the pyrrole, which was first distilled under reduced pressure for high purity and stored at room temperature in the absence of light prior to use. Distilled water was used throughout the experiments. The radiotracer Cd-109 (activity 3 μCi = 111 kBq, traceable in 10 ml of 4 M HCl solution) was purchased from Eckert and Ziegler Analytics, supplied in a flame sealed vial.

The sawdust sample (*Dryobalanops aromatic*) was kindly provided by the wood section of the Mechanical workshop of the Department of physics, University of Malaya. The *Dryobalanops aromatic* tree, also known as camphor tree, is a very heavy hardwood and probably the tallest found in the Malaysian forest, and it is commonly used as a building material in Southeastern Asia. The solid materials on the collected sawdust sample were hand-picked to separate the sawdust from dirty solid particles before washing with distilled water to remove dust-like impurities from the surface. The clean sawdust was dried at 50°C for 48 h in an oven. The dried sample was sieved to fine particle sizes (0.125–0.212 mm) using US standard stainless steel sieve series (A.S.T.M. E–11 Specifications, Dual Mfg. Co.).

### 2.2 Synthesis of polypyrrole coated sawdust composites and characterization

The synthesis of the polypyrrole was performed in aqueous solution via chemical polymerization at room temperature and atmospheric pressure. The oxidant FeCl_3_.6H_2_O was added drop wise to the pyrrole solution with the monomer to oxidant mole ratio of 1:1 to form polypyrrole. The mixture was shaken for 4 hours and a black polypyrrole polymer product was obtained. The mixture was thoroughly washed with distilled water to remove the excess oxidant. The black polypyrrole product was then dried in an oven.

In the preparation of PPy/SD composite, the polypyrrole was synthesized directly on the surface of the sawdust (SD) following previous procedures [16] but with little modification. Here, the already processed sawdust was first added to the pyrrole solution in requisite amount and agitated for 12 h. The pyrrole-soaked sawdust was separated from the solution by filtration and then dispersed into the oxidant solution. This was further agitated for another 4 h for complete polymerization. The black precipitate was separated from the solution and washed for 4–5 cycles with distilled water before drying in the oven at 50°C for about 48 h. In each case, the yield of the prepared powder was more than 80%.

The sorbent characterization was performed by Brunauer, Emmett and Teller (BET), FESEM (Field emission scanning electron microscopy) and FTIR (Fourier transform infrared spectroscopy) techniques. The BET surface area and porosity were measured from the N_2_ gas-adsorption/ desorption isotherm at 77.4K using Micromeritics (ASAP 2020 and Tritar II 3020 Kr). The FESEM (S-4160, Hitachi, Japan) was operated at 2 kV accelerating voltage and 11.3 A emission current. The analysis by FTIR spectroscopy was done using a Perkin-Elmer Spectrum (Wavenumber: 500–4000 cm^-1^) on KBr pellets by press technique under uniform pressure.

### 2.3 Batch adsorption experiment

In this study, a ^109^Cd solution of 33.8–138.3 Bq/L was taken for the adsorption studies. 50 ml of the solution was contacted with 0.3 g PPy/SD under shaking at ambient temperature. The pH-meter was standardized using standard buffer solutions (pH values 4.0, 7.0 and 10.0). The solution pH was adjusted using 0.1 M NaOH and HCl solutions to pH 3.7–9.0 for the equilibrium pH studies. Effect of sorbent dose was investigated by contacting 0.3 to 1.0 g of the sorbent with the ^109^Cd solution. Effect of interfering metal ions such as sodium and potassium ions was also investigated on the adsorption. Finally, the effect of temperature on sorption of ^109^Cd was studied by varying the temperature of ^109^Cd solution in the range of 25–50°C. The radioactivity (Bq L^–1^) of ^109^Cd was analyzed using γ–ray spectrometry by means of a p–type coaxial HPGe detector having 28.2% relative efficiency and an energy resolution of 1.67 keV –full width at half maximum (FWHM) at the 1332.5 keV peak of ^60^Co, which was coupled to a desktop based multi–channel analyzer card system for data acquisition [19,20] The removal efficiency and amount of radioactivity adsorbed (Bq/g) by PPy/SD were calculated using the eqs (1) and (2), respectively:

$$\text{RE}=\;\frac{{\text{C}}_{0}-C_{e}}{C_{0}}\times 100$$ 1

$$q=\;\frac{C_{0}\;\times \;V_{i}\;-C_{e}\;\times \;V_{f}}{m}$$ 2

Where RE is the removal efficiency (%), C_0_ is the initial radioactivity of the ^109^Cd solution and C_e_ is the radioactivity of the supernatant solution, m is the amount of the adsorbent, q is the adsorbed amount of radioactivity (Bq/g) and V_i_ and V_f_ are the volumes of solution before and after adsorption. Both isotherm and kinetic models were evaluated for the adsorption process.

## Results and Discussion

### 3.1 Characterization of PPy/SD composites

**Fig 1:**
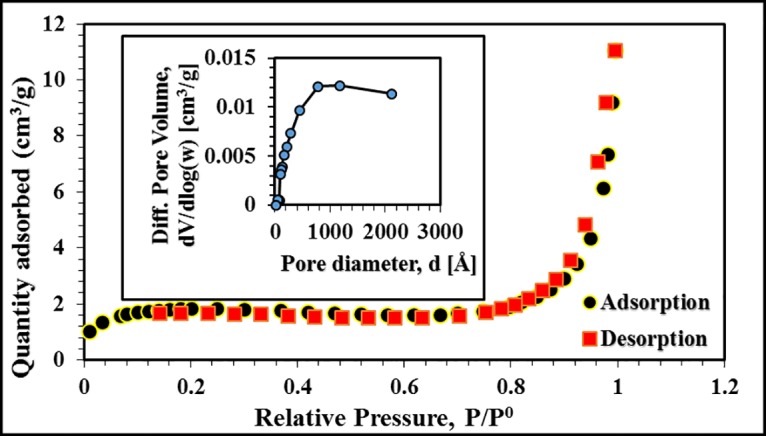
N_2_ adsorption-desorption isotherms and pore size distribution (inset) of PPy/SD composite.

The surface area measurement of PPy/SD composite in dried powder form was carried out by BET technique. Pore diameter of the PPy/SD composite was 39.48 nm and the total pore volume (single point adsorption volume of pores less than 3253.07 Å with p/p_0_ = 0.994) was 0.017 cm^3^/g before ^109^Cd adsorption. The BET and Langmuir surface areas measured were 7.06 and 8.48 m^2^/g, respectively. The nitrogen adsorption–desorption isotherm curve (Type II curve) with pore size distribution (inlet) was obtained as shown in Fig 1.

**Fig 2:**
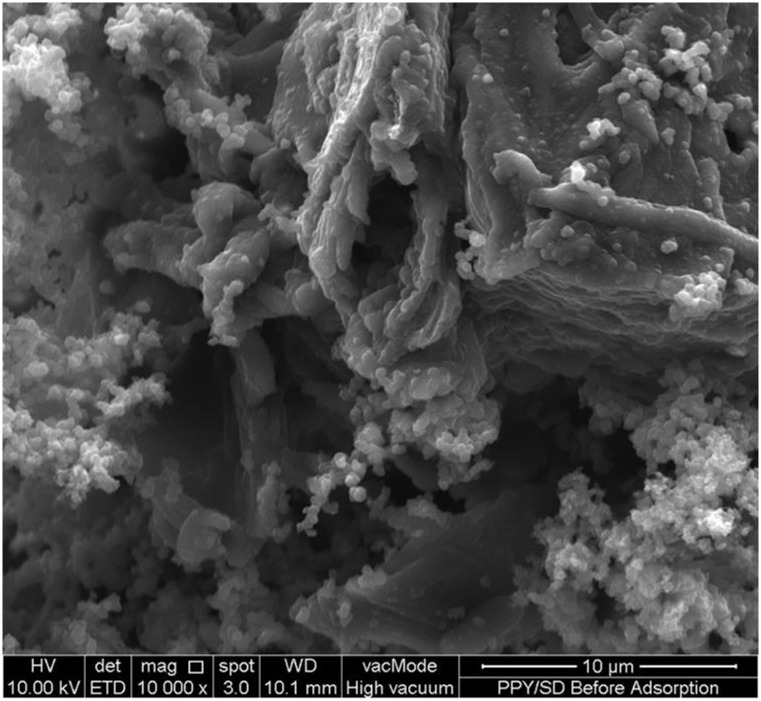
(a) FESEM image of PPy/SD composite.

The surface morphology of PPy/SD composite before Cd-adsorption was investigated using FESEM (Fig 2). The FESEM image shows that the pyrrole monomer was successfully polymerized on the SD with some PPy precipitate dispersed on the surface of the PPy/SD composite. Formation of PPy precipitate has been reported to be pronounced at higher concentration of pyrrole [17].

**Fig 3:**
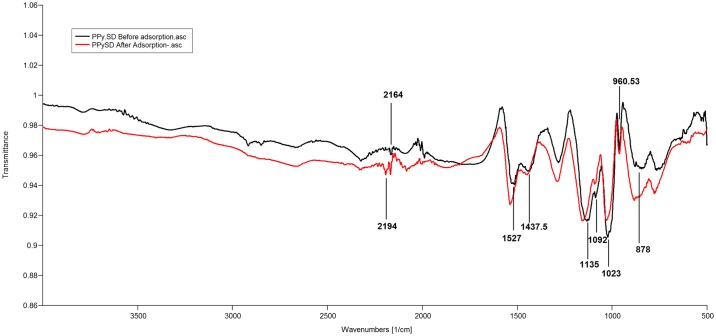
FTIR spectra of PPy/SD composite.

The structure of PPy/SD composite was determined by FTIR spectra to identify the functional groups involved in adsorption of cadmium, and a comparison between the FTIR spectra before and after cadmium loading was done (Fig 3). Some of the notable bands in the PPy/SD composite are found at 2164 cm^-1^ (due to the C-H stretching vibration), 1527 cm^-1^ (due to antisymmetric and symmetric vibration of pyrrole ring), 1437.5 cm^-1^ (due to C-O streching of carboxyllic groups, due to C = C streching of benzenoid ring), 1135 cm^-1^ (due to C-N streching), 1092 cm^-1^ (due to C-O bond streching of COOH), 1023 cm^-1^ (due to symmetrical C-H in-plane bending and N-H in-plane deformation), 960.53 cm^-1^ (due to C-H out-plane bending vibration) and 878 cm^-1^ (due to OH alcohols and aliphatic ethers). These bands showed some shifts after adsorption and in addition, a new band was also noticed at 2194 cm^-1^ confirming the complexation of cadmium ions with the functional groups. In particular, the shift in the band from 1023–1027 cm^–1^ reflects the deprotonation of the N-H groups, followed by the coordination of nitrogen to the metal ions [15].

Note that there was no shift in the band between 980 and 940 cm^-1^ which has been assigned to phenolic groups (OH), and this could be due to the fact that the functional groups did not contribute to ^109^Cd adsorption by ion-exchange mechanism.

### 3.2 Effects of adsorption conditions

In this investigations using 0.3 g of the adsorbent, it was found that the PPy alone has the capacity to remove 60% of the cadmium, while the PPy/SD composite removed about 99% of the cadmium from solution. This may be due to the synergic effects of the two components on the polymer composite, leading to higher adsorption compared to the pure polypyrrole. As a result, the rest of the adsorption study was conducted using the as-prepared PPy/SD composite.

**Fig 4:**
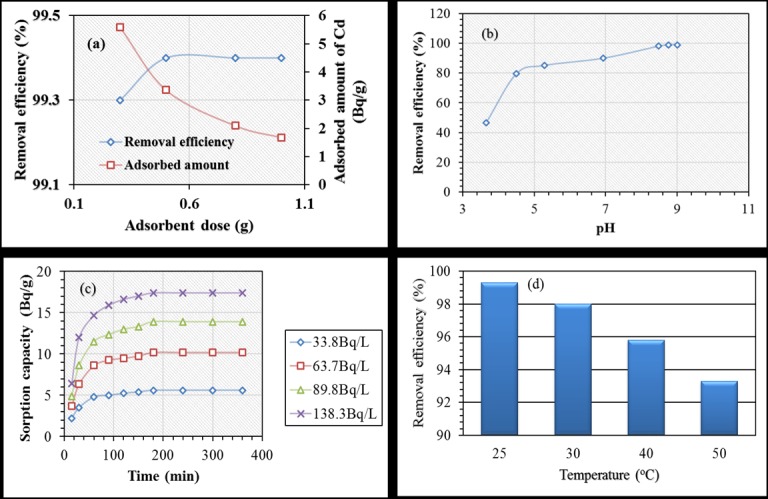
Effect of (a) PPy/SD dose, (b) Solution pH, (c) Contact time and (d) Temperature on cadmium adsorption.

The effect of adsorbent dose was investigated on removal efficiency and adsorbed amount of radioactivity by contacting 0.3 to 1 g PPy/SD with 50 ml of solution containing 33.8 Bq/L while solution was maintained at pH 8. The results are presented in Fig 4a. As shown, the removal efficiency was relatively increased and amount sorbed decreased as adsorbent dose increased. Decreasing of adsorbed amount can be explained in term of increasing unsaturated sorption sites due to increase in the amount of adsorbent for a fixed initial radioactivity and therefore leads to reduction in the amount retained per unit mass of adsorbent. When 0.3 g of PPy/SD composite was added to the solution, cadmium was removed by about 99.3%, but increase was not observed above 0.3 g. On the other hand, the amount adsorbed was about 5.6 Bq/g for the adsorbent dose of 0.3 g and was further reduced at higher doses, indicating maximum adsorption capacity at 0.3 g. Accordingly, the rest of the experiments were performed using a PPy/SD dose of 0.3 g.

Since a change in the pH of solution could affect not only the surface charge of the adsorbent but also the degree of ionization of the functional groups and chemical speciation of adsorbate during adsorption [17,18], the effect of solution pH was performed for each test by varying the initial pH of solution from 3.7 to 9.0. As shown in Fig 4b, there was low removal in the acidic pH range (46.7%). As the pH increased from pH 5.3 to 8.0, the adsorption increased to its highest (> 99%). This trend was continued until pH 9. However, further increase in pH beyond 9 was not followed due to the possible precipitation of CdCO_3_ and / Cd(OH)_2_ at higher pH (>9). It is clear from the behavior of adsorption of ^109^Cd that as the pH lowers, the surface of the adsorbent becomes positively charged due to the increase of hydronium (H_3_O^+^) ion concentration in the aqueous phase competing for the sorbent adsorption sites, leading to less uptake of cadmium. However, as the pH increases, the concentration of hydronium (H_3_O^+^) ion is reduced, leaving the sorption sites vacant for cadmium ions and, hence, results in a higher uptake of cadmium. The adsorption behavior of the composite adsorbent for cadmium ions in the solution could also be explained in terms of reactive functional groups present in each component of the adsorbent. In the sawdust, various chemical binding agents such as cellulose chains and polar groups (which include alcohols, aldehydes, ketones, hydroxides present in lignin) may be influenced by the variation of solution pH [21]. This was also suggested by others who have studied cadmium adsorption on sawdust [3,22]. In the case of polypyrrole in the doped state, the surface functional groups (such as the amine groups) can be greatly influenced with solution pH [13]. As a result, lower pH can induce more protonation of surface amine sites which could result in less electrostatic interactions between the polymer composite and cadmium ions. Conversely, as solution pH increased, deprotonation of amine groups occurring in the polymer matrix resulted in the creation of vacant sites for the adsorption of cadmium ions. Other mechanisms such as π-π stacking interactions originating from the conjugated polymer could also contribute to the adsorption process.

The effect of contact time is an important parameter in studying adsorption processes. At different ^109^Cd radioactivity, the relationship between the contact time and the amount of ^109^Cd adsorbed is presented in Fig 4c. It showed that fast adsorption was possible within 2 h, which then slowly reached equilibrium in 3 h. Contact time was extended to 6 h to ensure equilibrium. Rapid adsorption within the first 2 h can be related to the vacant sites of the sorbents available for adsorption. As time increases, adsorption may progress by slow diffusion of the remaining adsorbate into the bulk of the adsorbent [4,23]. It was found that the removal of ^109^Cd increased from 75.4 to 99.3% when decreasing the initial radioactivity from 138.3 to 33.8 Bq/L. Also, the amount of adsorption per gram of the adsorbent was found to increase with contact time at all initial radioactivities until equilibrium is attained in 3 h. Moreover, raising the initial ^109^Cd radioactivity from 33.8 to 138.3 Bq/L allows the increase of amount adsorbed from 5.59 to 17.38 Bq/g possibly due to the increasing driving force to overcome the resistance to mass transfer of activity of radionuclide from the aqueous phase to the solid phase. This also explains the reduction in the removal efficiency for a constant dosage of adsorbent at higher radioactivity due to saturation of the available sorption sites with increasing radioactivity. However, sorption capacities could be higher for higher activity of ^109^Cd but it was avoided here because of the poisonous and radiation health hazards of ^109^Cd. Similar equilibrium contact time of 3 h was reported in the literature for Cd(II) adsorption onto biochar [24]. Meanwhile, shorter contact time of 12 min was reported using PPy for Cd(II) adsorption [15], and chemically treated/untreated sawdust for cadmium ion removal [25].

The temperature dependent studies were carried out from 25 to 50°C at pH 8.0, initial activity concentration of 33.8 Bq/L, 0.3 g adsorbent dosage, 3 h of contact and 250 rpm shaking speed. Results show that an increase in temperature corresponds to a decrease in the removal efficiency (from 99 to 93%) (Fig 4d). This suggests the exothermic nature of cadmium sorption by PPy/SD composite, which is common with the most of gas adsorption processes. Beside this, since increase in temperature increases the rate of diffusion of ions, it is possible here that the diffusion of cadmium ions through PPy/SD composite is not affected by a change in temperature. This was the similar case with the diffusion of cadmium ions through chitin previously reported [26]. It was also reported that when cadmium was removed by sawdust of *Pinus sylvestris*, an increase in temperature decreased the removal efficiency [3], but it was increased in case of recombinant *Escherichia coli* [27].

For the effect of competing metal ions in adsorption of Cd, previous studies have reported negligible effects of lead and copper but zinc and manganese inhibited cadmium selectivity by many sorbents including recombinant *Escherichia coli* [1,27]. As a result, the effect of Na and K ions was investigated which revealed that the uptake of ^109^Cd was not favorable in the presence of both metal ions even at low concentration. At 0.1 M of both metal ions, the removal efficiency decreased from 99.3 to 51%, and was found much lower (~20%) at 0.2 M but no significant adsorption occurred when the metal ions concentration increased above 0.2 M. The selective adsorption of Na^+^ and K^+^ ions over Cd^2+^ ions may be related to their high chemical reactivity and stronger complexation towards solid surface compared to Cd^2+^ ions.

### 3.3 Adsorption kinetic models

**Fig 5:**
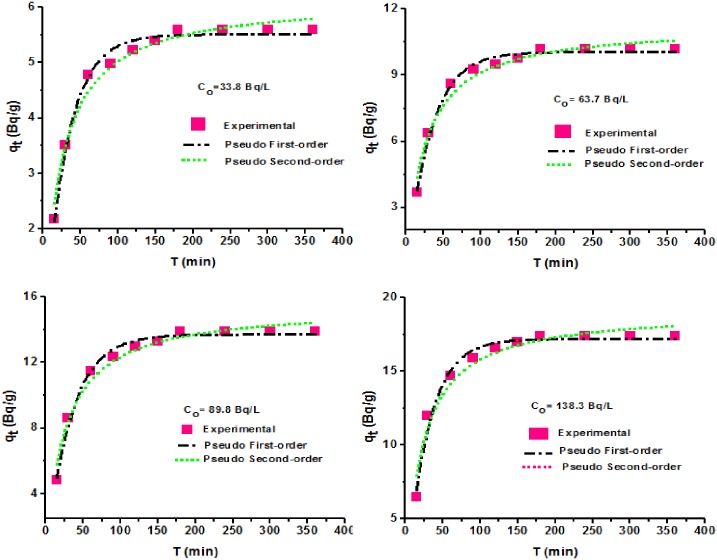
Adsorption kinetic models for ^109^Cd adsorption by PPy/SD composite.

Adsorption of a given solute on a solid phase can proceed by different mechanisms depending on the heterogeneity of reactive sites and the physico-chemical conditions under which the adsorption takes place. The knowledge of sorption mechanism for a particular sorbate is important to effectively describe the efficiency and design future feasibility of the adsorption at large scale. In the current study, two commonly employed kinetic models namely pseudo first-order and pseudo second-order models were used to predict the sorption mechanism and the rate controlling steps during the sorption process. The non-linear expressions for the two models were used to estimate the adsorption parameters as listed in Table 1 and Fig 5. As shown, the two models yield good correlations to the kinetic data. However, comparing the correlation coefficients (R^2^) of the two models and the obtained results to the kinetic data showed that the pseudo first-order fits best than the pseudo second-order kinetics. The fitting of the kinetic data to the pseudo first-order model was an indication that the rate controlling reaction could be a physical process. Consequently, it indicates that the adsorbate can be recovered from the spent adsorbent without losing its identity by desorption [28]. However, the present study did not investigate desorption of cadmium ions but will be considered in the ongoing studies and that will be reported in due time. The literature revealed that previous studies have reported the good applicability of the pseudo first-order model for the cadmium ions adsorption onto the polypyrrole [15]. Other studies on the sorption of cadmium ions onto sawdust of *Pinus sylyestris* [3], chitin [26] and natural ball [29] reported the fitting of kinetic data to the pseudo-second order kinetics.

**Table 1:** Kinetic model equations and parameters related to the adsorption of cadmium radionuclide onto PPy/SD composite.

Kinetic model	Equation	Non-linear form	Parameter	Initial cadmium-109 activity (Bq/L)			
				33.8	63.7	89.8	138.3
**Pseudo-first order model**	i	ia	**Experimental, qexp (Bq/g)**	5.59	10.19	13.90	17.38
			k_1_ (min^−1^)	0.0326	0.0317	0.0302	0.0345
			Calculated, q_cal_ (Bq/g)	5.51±0.052	10.05±0.085	13.71±0.133	17.19±0.189
			R^2^	0.988	0.991	0.989	0.983
**Pseudo-second order model**	ii	iib	k_2_ (g/Bq min)	0.0072	0.0038	0.0026	0.00248
			Calculated, q_cal_ (Bq/g)	6.16±0.12	11.27±0.25	15.44±0.34	19.13±0.46
			R^2^	0.975	0.970	0.973	0.957

i $\frac{{dq}_{t}}{dt}\;=\;k_{1}(q_{e}-q_{t})$; ia $q_{t}\;=\;q_{e}(1-e^{\left(-k_{1}t\right)})$; ii $\frac{dq_{t}}{dt}\;=\;k_{2}{\left(q_{e}-q_{t}\right)}^{2}$; iib $q_{t}\;=\;\frac{k_{2}q_{e}^{2}t}{1+k_{2}q_{e}t}$.

### 3.4 Adsorption isotherms

The relationship existing between the sorbate in the liquid and solid phase at equilibrium is very important in order to understand the mechanism involved in the adsorption process and to optimize the use of adsorbent materials. The amount of sorbate sorbed to the solid material relative to the concentration in the liquid phase at equilibrium is referred to as an adsorption isotherm. We employed the models of Langmuir, Freundlich and Temkin to predict the sorption parameters from the experimental data.

The Langmuir model describes the monolayer adsorption of solute on the homogeneous surface without interactions between the sorbed molecules. The model is given by:

$$q_{\text{e}}=q_{\text{m}}\;\frac{{\text{bC}}_{\text{e}}}{1\;+{\text{bC}}_{\text{e}}}$$ 3

Where q_e_, q_m_ and b are the adsorption capacity (Bq/g) corresponding to cadmium radioactivity (at equilibrium C_e_), maximum adsorption capacity of ^109^Cd (Bq/g) and Langmuir adsorption constant (L/g), respectively.

The essential features of the Langmuir adsorption isotherm can be expressed as a dimensionless separation factor R_L_, which is derived as follows [21]:

$$R_{L}=\frac{1}{1+bC_{O}}$$ 4

The R_L_ value indicates the isotherm shape favourable (0 < R_L_ < 1) or unfavourable (R_L_ > 1) for adsorption.

The Freundlich model assumes a distribution of sorption active site and their energies [30]. The model is expressed as given below:

qe=KFCe1n 5

Where, K_F_ is known as affinity coefficient which indicates sorption capacity and, if adsorption process is exothermic, the affinity coefficient (K_F_) decreases with a rise in temperature. The other parameter “n” denotes the intensity of adsorption process and the distribution of active sites. If n < 1, it means bond energies increase with surface density and if n > 1, bond energies decrease with the surface density.

The third isotherm model is Temkin, which also suggested that the heat of adsorption of all the molecules would decrease linearly with the increase in coverage of the sorbent surface due to some indirect interactions between the adsorbed cadmium ions. This model is expressed as given below:

$$q_{e}=\frac{RT}{b}In\left(AC_{e}\right)$$ 6

Where, $B\;=\;\frac{RT\;}{b}$ and A are constants related to the heat of adsorption and the maximum binding energy at equilibrium.

**Fig 6:**
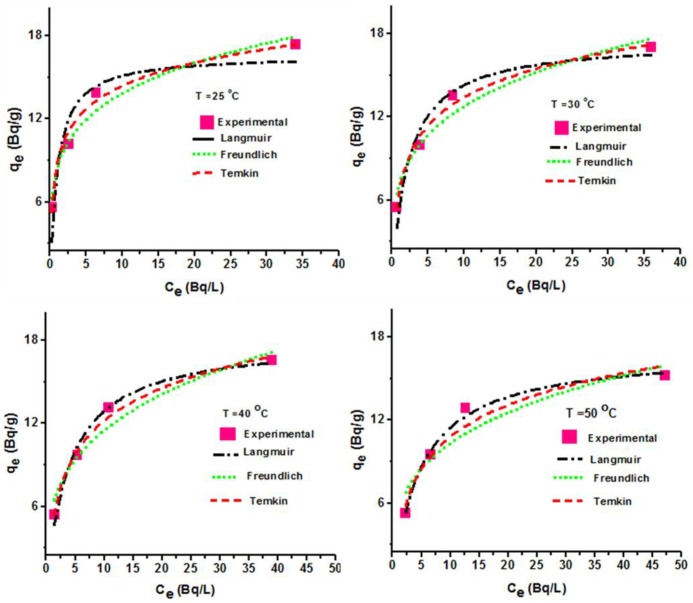
Adsorption isotherm models (a) Langmuir model, (b) Freundlich model and (c) Temkin model.

The Langmuir, Freundlich and Temkin sorption parameters estimated from the isotherm plots (Fig 6) at different temperatures and the corresponding correlation coefficients are presented in Table 2. As shown, all the isotherm models offer good correlation factors for radioactive cadmium adsorption, despite the low radioactivity involved in the study. This suggests that PPy/SD composite is efficient to treat low radioactive waste-effluents especially from secondary sources such as radioactive laundry wastewater if the effects of the competing metal ions such as sodium, potassium etc are negligible. In addition, the decrease in the values of b (0.998–0.205 L/g), n (4.747–2.88) and A (38.19–2.592) obtained from the Langmuir, Freundlich and Temkin models, respectively, as temperature increases, indicates the exothermic nature of the adsorption, and that the bond energies decrease with surface density. Also, the range of R_L_ values (0.016–0.071) supports that the adsorbent can be favourably exploited in practical application. Meanwhile, despite that the Freundlich model can provide information on the distribution of active sorption sites and their energies, it cannot predict for sorbent surface saturation by the adsorbate due to its failure to linearize as adsorbate concentration tends to infinite dilution, which is the case of sorption [28]. Besides, the three models do not have same fitting parameters and hence, it is not advisable to conclude that the adsorbate/adsorbent interactions can be described by a particular mechanism solely on the basis of good data fit to one model over another [28]. Therefore, we can only consider the application of these models for mathematical representation of the sorption equilibrium for a given metal-ion concentration range and not for the adsorption behavior of cadmium ions onto complex organic system such as polypyrrole/sawdust composite. In spite of these limitations, these models still provide information on adsorption capacities and differences in uptake of adsorbate between various species [26].

**Table 2:** Summary of the Langmuir, Freundlich and Tempkin Isotherm Constants, Separation Factor (R_L_) and Linear (R^2^) Regression Coefficients.

Model	Parameter	Temperature			
		25°C	30°C	40°C	50°C
**Langmuir**	q_m_ (Bq/g)	16.622	17.513	18.064	17.026
	b (L/g)	0.998	0.446	0.248	0.205
	R_L_	0.0156	0.0341	0.0594	0.0707
	R^2^	0.792	0.926	0.980	0.987
**Freundlich**	K_F_ (L/g)	8.529	7.107	5.852	5.414
	n	4.747	3.938	3.404	3.563
	R^2^	0.951	0.938	0.922	0.828
**Temkin**	A	38.18	9.145	3.466	2.592
	B	2.420	2.971	3.435	3.317
	R^2^	0.976	0.984	0.983	0.931

Comparison of the cadmium uptake by PPy/SD composite with other adsorbents was made. As shown in Table 3, sorption capacities varied widely for different adsorbents which is due to different experimental conditions under which the adsorption has been investigated. The fact that the radioactivity level investigated in the present study is low and adsorption kinetics was rapid compared to most of the literature, makes the PPy/SD a potential adsorbent for consideration to treat low-level radioactive waste effluents.

**Table 3:** Comparison of cadmium adsorption by PPy/SD with the literature.

Adsorbent	Equilibrium pH	Temperature (°C)	Maximum capacity	Reference
PPy/SD composite	8	25	16.622 Bq/g	This study
PPy	6.6	25	67.92 mmol/g	[15]
Chitin	5.70–6.02	25	14.706 mg/g	[26]
PANI/SD	6	20.5	430 mg/g	[31]
Magnetic hydroxyapatite nanoparticles	5	25	1.964 mmol/g	[32]

## Conclusion

Polypyrrole conducting polymer modified with sawdust of *Dryobalanops aromatic* tree has proven to be a good adsorbent for ^109^Cd radionuclide from low level radioactive effluents. The removal efficiency of as–prepared PPy/SD composite was found to be about 99.3% which was attained within 3 h of contact at 25°C. Fast adsorption of ^109^Cd from the aqueous solution suggested that PPy/SD is a promising adsorbent material for the removal of ^109^Cd from waste effluents. The kinetic study and FTIR analysis reveal that the adsorption of cadmium by PPY/SD composite is mainly due to the metal ion coordination with nitrogen of polypyrrole and the metal complexation formation with functional groups of sawdust. Considering the low cost of the precursor’s materials and the toxicity of radioactive metals, polypyrrole synthesized on the sawdust of *Dryobalanops aromatic* could be used as an efficient adsorbent for the removal of ^109^Cd from low level cadmium radionuclide-containing effluents.
